# Red Ants

**Published:** 1873-02

**Authors:** 


### RED ANTS

Are a great pest to housekeepers in certain localities. Whether it is "healthy" to eat them, is a question which has not yet been decided; the idea, however, has emetic tendencies, hence it is rather better to keep them out of our sugar and cake and other nice things, which they are pretty sure to affect, to avoid which, put each foot of your refrigerator in a bowl, and keep it half filled with water—the bowl, not the refrigerator; in the first place, they can't well climb up into the bowl, and if they could, they could not swim across the water.

Pulverized borax, innocuous to man, seems to have the power of keeping them off any shelf on which it is freely sprinkled, or dampen a sponge, work into it most thoroughly pulverised loaf sugar, lay it on the place frequented, over night; in the morning it will be loaded with ants, then put the sponge in boiling water; repeat as often as necessary.

